# Ultra-compact high efficiency and low crosstalk optical interconnection structures based on inverse designed nanophotonic elements

**DOI:** 10.1038/s41598-020-68936-w

**Published:** 2020-07-20

**Authors:** Zikang Li, Guofeng Li, Jie Huang, Zhenrong Zhang, Junbo Yang, Changming Yang, Yang Qian, Wenjie Xu, Huimin Huang

**Affiliations:** 1 Guangxi Key Laboratory of Multimedia Communications and Network Technology, School of Computer, Electronics and Information, Guangxi University, Nanning, 530004 China; 2 Center of Material Science, National University of Defense Technology, Changsha, 410073 China; 3 State Key Laboratory on Advanced Optical Communication Systems and Networks, Peking University, Beijing, 100871 China

**Keywords:** Nanoscience and technology, Optics and photonics

## Abstract

In this paper, we combine inverse design concept and direct binary search algorithm to demonstrate three ultra-compact high efficiency and low crosstalk on-chip integrated optical interconnection basic devices in the entire wavelength range of 1,400–1600 nm based on silicon-on-insulator platform. A 90-degree waveguide bend with a footprint of only 2.4 × 2.4 μm^2^ is designed, whose transmission efficiency up to 0.18 dB. A waveguide crossing with a footprint of only 2.4 × 2.4 μm^2^ is designed, which can provide insertion loss of less than 0.5 dB and crosstalk (CL) of lower than − 19 dB. A same direction waveguide crossing with footprint of only 2.4 × 3.6 μm^2^ is designed, which can provide the insertion loss of less than 0.56 dB and the crosstalk of lower than − 21 dB. Then, we use them to form several ultra-compact optical interconnect basic structures and performed the simulation calculation. They overall achieve high performance. This will significantly improve the integration density.

## Introduction

The concept of integrated photonics was introduced in 1969 by Miller^1^, which made a great contribution to the on-chip integrated optical interconnection. On-chip integrated optical interconnection is an emerging technique for large capacity data communications. The waveguide bends and crossings are one of the most critical components of optical interconnection. There are many waveguide bends and crossings designed by conventional approaches. A 5 μm radius 90-degree circular bend with 400 × 220 nm silicon strip waveguide was designed^2^. Although the loss of the circular bend is 0.023 dB in FDTD simulation, the design method need a lot of complicated artificial adjustment experiments. A 90-degree partial Euler bends with an effective radius of 50 µm was fabricated on a silicon nitride photonic^3^. The bend loss was within 0.2 dB, but it was only limited to the wavelength of 850 nm and had a large footprint. A 275 µm radius circular bend was designed using a silicon waveguide^4^, which achieved very low loss. Unfortunately the length of the radius and waveguide is too long. Bogaerts et al.^5^ proposed a 90-degree adiabatic bend for silicon waveguides. The total bend loss was 0.001 dB. However, the curve radius and circular wire section were up to 5 µm and 25 µm, respectively. Other waveguide bends with 2µm^6^, 4µm^7^, 10µm^8^ radius were designed using a straight waveguide. Although the loss was below 0.3 dB loss, they had a large footprint and need a very long waveguide. Various studies have also been conducted on waveguide crossings. A low-loss waveguide crossing using the self-imaging properties of multimode interference (MMI) structures was designed^9^, which achieved a loss of only 0.009 dB. But, the length is also fairly long (> 140 µm in the case). A multimode-interference (MMI)-based crossing in high-index-contrast silicon wire waveguides was reported, which had an MMI crossing of ~ 0.4 dB insertion loss^10^. However, its large 13 × 13µm^2^ footprint may be an issue for dense integration. Recently there have been reports attempting to further reduce the footprint with successful demonstrations with a 6 × 6µm^2^ ^11–14^. Note that − 1.7 dB insertion loss was achieved with only − 40 dB cross talk^11^. Insertion loss was lower than 0.2 dB and crosstalk loss was below 40 dB in a broad bandwidth of 20 nm^12^. Average insertion loss was 0.18 ± 0.03 dB and crosstalk was − 41 ± 2 dB^13^. The waveguide crossing only operated at 1550 nm and 1310 nm, the results showed transmission insertion loss of − 0.028 ± 0.009 dB only for the 1550 nm device and − 0.017 ± 0.005 dB only for the 1310 nm device. Both crossings show crosstalk lower than − 37 dB^14^. Another waveguide crossing showed an insertion loss of 1.82 dB as well as a crosstalk of <  − 18 dB from 1,510 to 1600 nm^15^. However, the footprint of the device was as compact as 21 × 21 μm^2^. A dual-mode waveguide crossing is proposed. The characterization results for the fabricated device show that low insertion loss below 1.5 dB and low crosstalk below − 18 dB can be achieved^16^. But, it must need two waveguide crossing to achieve the function. Han et al.^17^ used particle swarm optimization algorithm to design a SOI waveguide crossing with a footprint of only ~ 1 × 1 μm^2^. Although it has an insertion loss < 0.175 dB and crosstalk around − 30 dB for the C-band, the designed method needs complex calculations and long-time simulation, and the number of iterations is set 500. A waveguide cross was designed that insertion loss and crosstalk were less than 0.6 dB and − 24 dB from 1530 to 1590 nm, respectively^18^. However, the fabricated device still occupied a footprint of 4.8 × 4.8 μm^2^. Although traditional methods have been used to design devices with good performance, the footprint is always large that has a negative impact on dense integration, and the design of such devices rely heavily on the intuition and experience of the designer and cannot manually achieve a full-parameter space design. Therefore, we should use other methods to design waveguide bends and crossings. This method must be simple and feasible. It should greatly reduce the footprint of the device and achieve superior high performance.

In recent years, the inverse design method and DBS algorithm of ultra-compact silicon photonic devices have drawn more and more attentions^19–25^. The best-known institutions for inverse design research are the University of Utah and Stanford. The University of Utah achieved the inverse design of integrated nano-photonic polarization beam splitters by combining DBS algorithm and the current commercial software^19^. The Stanford used alternating directions method of multipliers (ADMM) to design a wavelength demultiplexer of 1300 nm/1500 nm^20^. For other optical device designs like flat optics and holograms, the inverse design also has a wide range of application^26–29^. Learning from their train of thought, Harbin Institute of Technology^30–32^, Huazhong University of Science and Technology^33–35^, National University of Defense Technology^36–40^ designed a variety of on-chip optical devices and achieved a good performance. One of the ways to realize inverse design is combining DBS algorithm and commercially available simulation software capable of implementing FDTD solutions, such as Lumerical FDTD Solutions^41^. Inspired by them, we used inverse design and DBS algorithm to design waveguide bends and crossings.

In this paper, based on SOI platform, we successfully demonstrated a 90-degree waveguide bend, a waveguide crossing and a same direction waveguide crossing in the entire wavelength range of 1,400–1600 nm by using DBS algorithm. Their footprints are only 2.4 × 2.4 μm^2^, 2.4 × 2.4 μm^2^, 2.4 × 3.6μm^2^ respectively. The transmission efficiency of the 90-degree waveguide is > 0.5 dB in the entire wavelength range of 1,440–1640 nm and up to 0.18 dB at some wavelengths. The waveguide crossing can provide insertion loss of less than 0.5 dB. The same direction waveguide crossing can provide the insertion loss of less than 0.56 dB. The measured crosstalk of the two crossings is lower than ‒19 dB and ‒21 dB respectively. Meanwhile, four kinds of optical interconnection modes are designed with the proposed bending and crossing, which realize the superior performance. Compared with traditional optical interconnection, the integration density and benefit various on-chip optical systems are greatly improved.

## Results

### Optical interconnection component

#### A 90-degree waveguide bend

**Figure 1:**
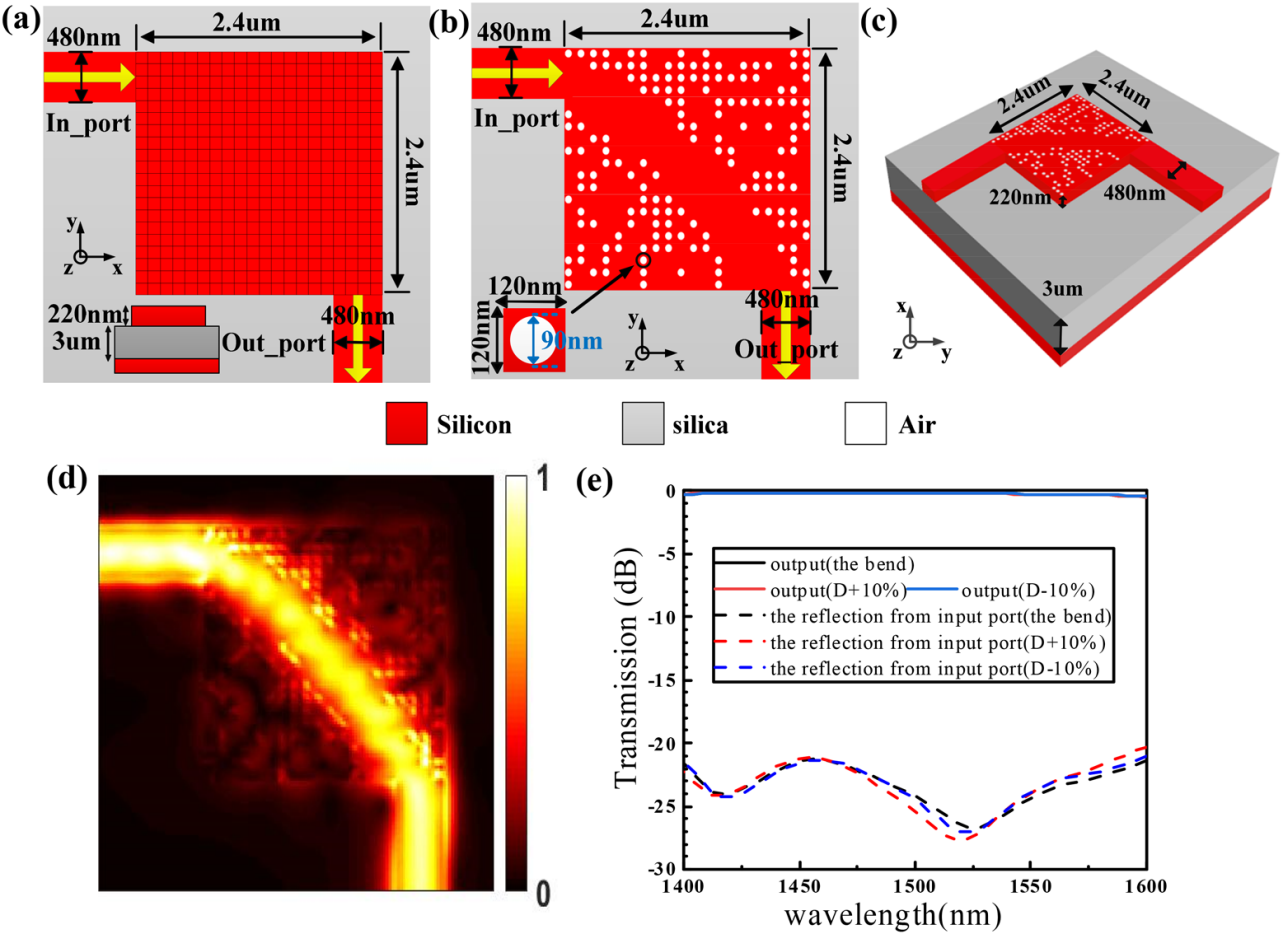
Design and simulation results of the 90-degree waveguide bend. (**a**) Initial silicon slab before design optimization. (**b**) Final optimized structure. (**c**) Isometric view. (**d**) Simulated optical field distributions. (**e**) Simulated normalized transmission spectra. Here, we have plotted the electromagnetic energy density *U* = ϵ|**E**|^2^ + μ|**H**|^2^. An animated version of **d** is available in Supplementary Video S1.

Based on the “design method”, we first design a 90-degree waveguide bend on a silicon-on-insulator (SOI) substrate with 220 nm silicon on top of 3 μm buried oxide. As shown in Fig. 1a–c, the computation domain is 2.4 × 2.4 μm^2^, where the optimization region is divided into 20 × 20 pixels and each pixel is a square of 120 nm × 120 nm with a central circular hole. The hole has a diameter of 90 nm and a depth of 220 nm, which can be fabricated with an electron-beam lithography (EBL) system (Vistec EBPG 5000 Plus).

Each pixel can occupy two states: silicon or air. The white and red regions of the pixel denote the etched area where no silicon remains on top of the oxide and the unetched area where the 220 nm silicon remains, respectively. At the beginning, all the pixel states are chosen to be silicon, and the excitation the TE_0_ mode in the wavelength range of 1,400–1600 nm. Then the state of each pixel is altered one by one, and the figure-of-merit (FOM) is inspected. Here the FOM is defined as:

1 $$ {\text{FOM}} = {\text{T}}_{{{\text{out}}\_{\text{port}}}} $$

where ${\text{T}}_{{{\text{out}}\_{\text{port}}}}$ is the transmission efficiency of the final structure at the wavelength of 1550 nm as the input light source is TE_0_ mode, which is calculated by 3D FDTD with 40 nm × 40 nm × 40 nm grid size and 8 layers PML. If the FOM is improved, the pixel state is retained. If not, the pixel state is reversed and the algorithm proceeds to the next pixel. One iteration ends after all the pixel states are inspected. Then the iterations continue until the FOM does not improve further.

We perform the device design and optimization using an eight-core desktop. For the 90-degree waveguide bend, it takes ~ 24 h on average to get the final results after 4 iterations. Given that fabrication variations can have a significant impact on real-world device performance, we characterize the same bends with a ± 10% variation in pillar diameter by simulation. The simulated optical field profile of the device when the continuous wave is launched from input port is shown in Fig. 1d. Figure 1e shows the calculated normalized transmission efficiency. The simulated normalized transmission efficiency of the bend is < 0.5 dB in the entire range of 1,400–1600 nm and up to 0.18 dB at some wavelength. As we can show, a ± 10% variation in pillar diameter doesn't affect largely on the 90-degree waveguide bend. It is seen from the Table 1 that the waveguide bend we designed has very low loss losses and a relatively small footprint compared with other waveguide bends. The method we used is feasible and auto-actuated.

**Table 1:** Comparison of the waveguide bends.

References	Bending area (μm^2^)	Propagation losses (dB)	Waveguide	Method	Note
This work	2.4 × 2.4	0.18	Etched silicon	DBS	Auto-actuated
^2^	5 × 5	0.023	~ 10 μm long waveguide	Variational optimization	Hand-actuated
^3^	50 × 50	0.2	~ 100 μm long waveguide	Numerical analysis	Hand-actuated
^4^	275 × 275	~ 0.1	~ 550 μm long waveguide	Beam-propagation	Hand-actuated
^5^	5 × 5	0.027	25 μm long waveguide	Manual adjustment	Hand-actuated
^6^	2 × 2	0.086	21 mm long waveguide	Manual adjustment	Hand-actuated
^7^	4 × 4	0.5	~ 8 μm long waveguide	Clothoid curves	Hand-actuated
^8^	10 × 10	0.02	~ 20 μm long waveguide	Micron-scale cores	Hand-actuated

#### Waveguide crossing

- A waveguide crossing

**Figure 2:**
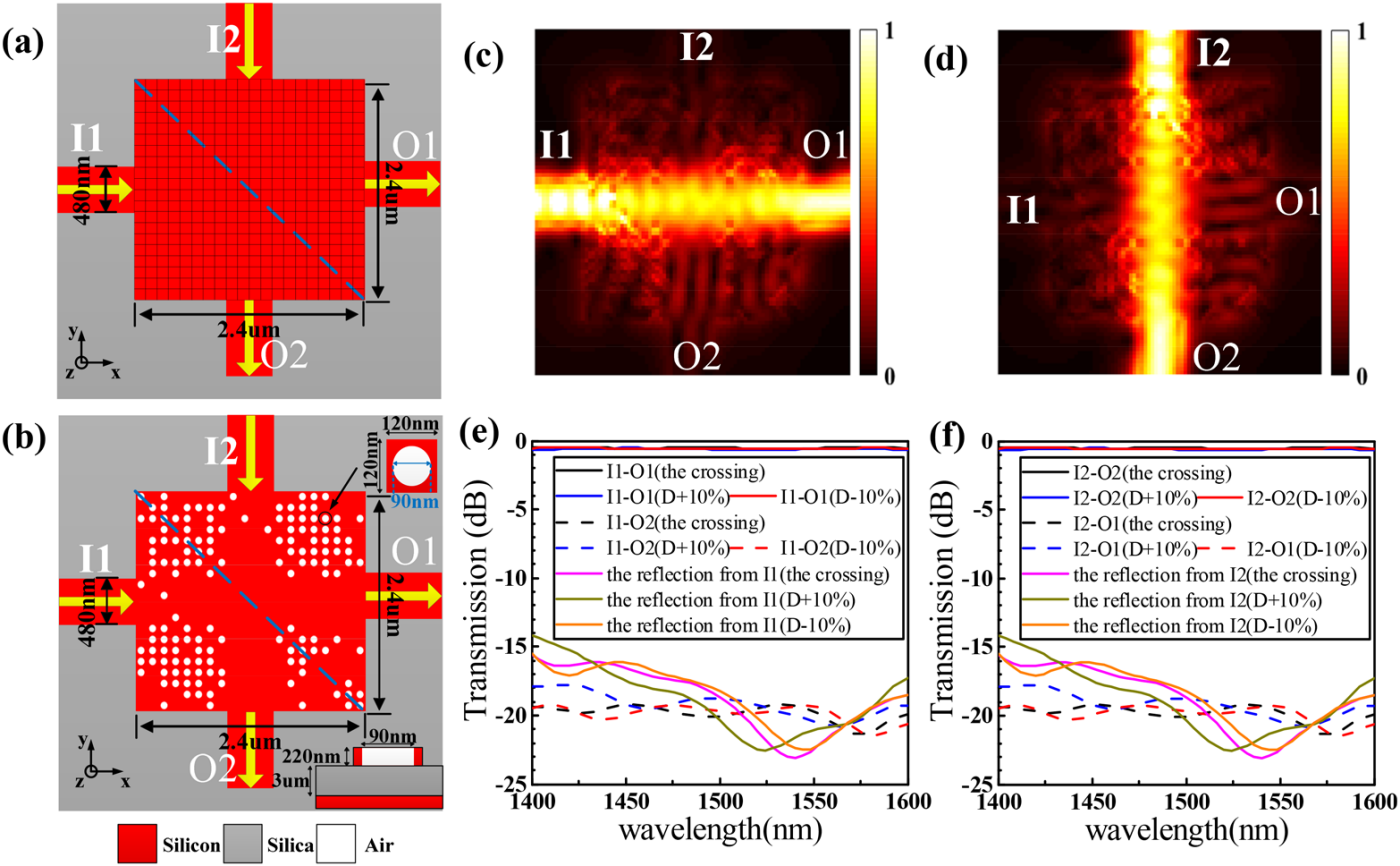
Design and simulation results of the waveguide crossing. (**a**) Initial silicon slab before design optimization. (**b**) Final optimized structure. (**c**), (**d**) Simulated optical field distributions. (**e**), (**f**) Simulated normalized transmission spectra. Here, we have plotted the electromagnetic energy density *U* = ϵ|**E**|^2^ + μ|**H**|^2^. An animated version of (**c**) is available in Supplementary Video S2.

The on-chip optical interconnection inevitably needs to address the waveguide cross-connect problem. Herein, we design a waveguide crossing with a footprint of only 2.4 × 2.4 μm^2^ using the same process as the bend, which has a quadrature symmetry. The structural schematic diagram of the device is described in Fig. 2a,b, the crossing has two input ports labeled as I1 and I2, and two output ports labeled as O1‒O2, respectively. The widths of the two input waveguides and the output waveguide are 480 nm.

This design region is also divided into 20 × 20 discrete pixels with minimum feature size of 90 nm. In the optimizing process, we use the TE_0_ mode in the wavelength range of 1,400–1600 nm as the only one light source placed at I1 port. The FOM of the crossing for inverse design is defined as:

2 $$ {\text{FOM}} = \left[ {{\text{T}}_{1} \left( {{\text{i}},{\text{j}} + 1} \right) > {\text{T}}_{1} \left( {{\text{i}},{\text{j}}} \right)} \right] \cap \left[ {{\text{T}}_{2} \left( {{\text{i}},{\text{j}} + 1} \right) \le {\text{T}}_{2} \left( {{\text{i}},{\text{j}}} \right)} \right] $$

where T_1_(i,j) and T_1_(i,j + 1) represent the average transmission of O1 port in the ith iterate of jth and (j + 1)th pixel, respectively. T_2_(i,j) and T_2_(i,j + 1) represent the average transmission of O2 port in the ith iterate of jth and (j + 1)th pixel, respectively. All the transmissions are calculated by 3D FDTD with 40 nm × 40 nm × 40 nm grid size and 8 layers PML. Actually, the first term in the right of Eq. (2) is used to optimize average insertion loss (IL) and the second one is employed to minimize the crosstalk (CT). If FOM = 1, it means IL is improved and CT descend, the pixel state is retained. If not, the pixel state is reversed.

It takes about 36 h after 4 iterations to get the optimized crossing on an eight-core desktop. To investigate the manufacturing error of the crossing, we test the same crossing with a ± 10% variation in pillar diameter. Figure 2c,d shows the simulated optical. The simulated ILs and CTs spectra at O1and O2 ports are plotted in Fig. 2e,f when the continuous wave is launched from input ports I1 and I2, respectively. The simulated CTs for the crossing and the same crossings with ± 10% diameter are < − 19 dB, < −  18 dB, < −  19 dB, respectively. The simulated ILs for all devices are ~ 0.5 dB from 1,400 to 1,600 nm wavelength range. A ± 10% variation in pillar diameter had nearly negligible impact on the IL. Due to a symmetric structure, the results obtained as the light source placed at I1 and I2 ports are consistent.

- A same direction waveguide crossing

**Figure 3:**
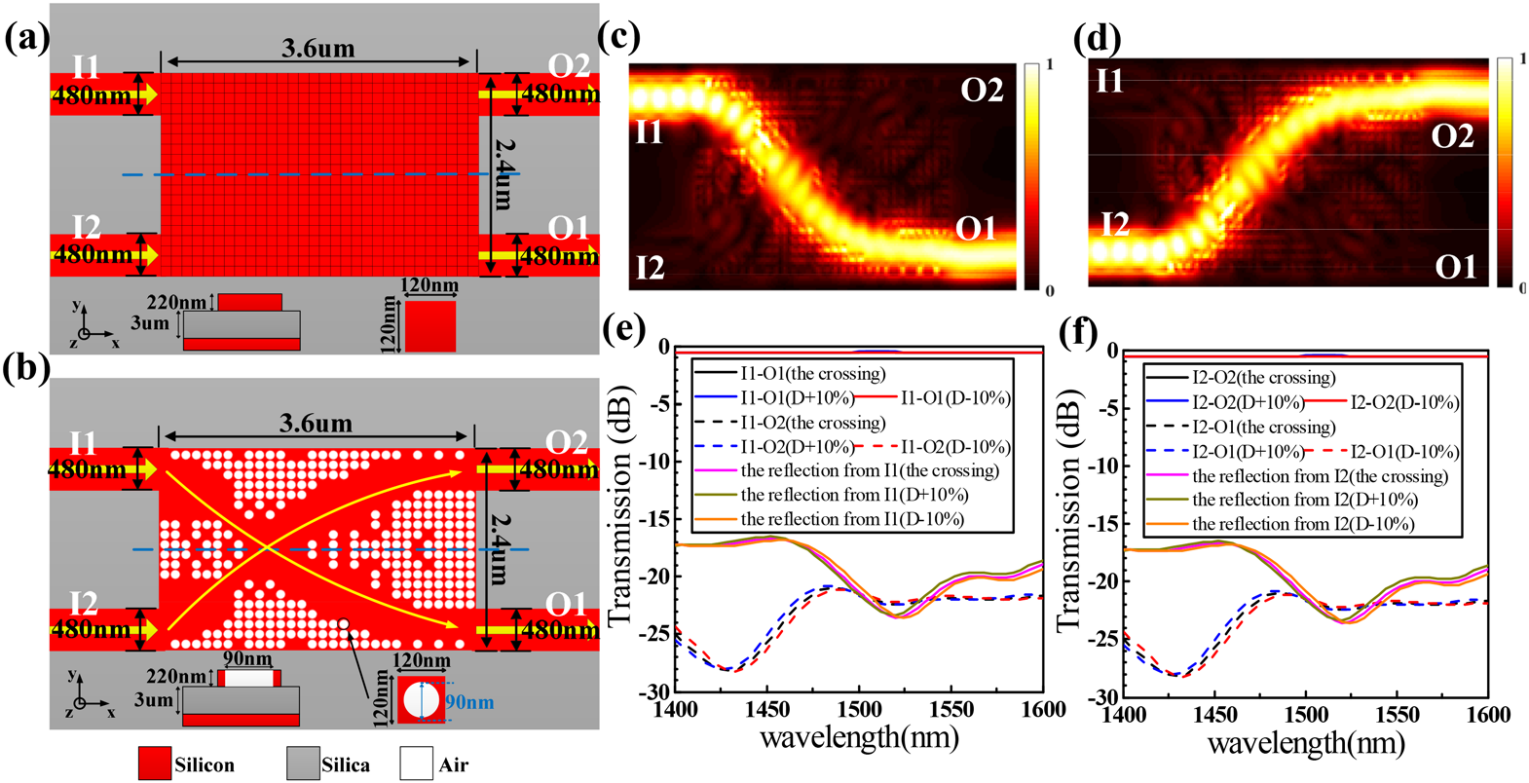
Design and simulation results of the same direction waveguide crossing. (**a**) Initial silicon slab before design optimization. (**b**) Final optimized structure. (**c**), (**d**) Simulated optical field distributions. (**e**), (**f**) Simulated normalized transmission spectra. Here, we have plotted the electromagnetic energy density *U* = ϵ|**E**|^2^ + μ|**H**|^2^. An animated version of (**c**) is available in Supplementary Video S3.

Here, we design another same direction waveguide crossing. As shown in Fig. 3a,b, the inverse design region composed of 20 × 30 discrete pixels occupies a compact footprint of only 2.4 × 3.6 μm^2^. The crossing is optimized using the same process as the waveguide crossing. The FOM is also defined as Eq. (2). It takes about 48 h after 5 iterations to get the final optimized structure on an eight-core desktop. Figure 3c,d shows the simulated optical and Fig. 3e,f shows the calculated ILs and CTs spectra when the continuous wave is launched from I1 and I2 ports, respectively. For the final crossing, the average IL is ~ 0.56 dB and the CT is <  − 21 dB within the wavelength range from 1,400‒1600 nm. Even though the diameter of the crossing changes with a ± 10% variation, the IL and CT curves are still quite consistent. Table 2 summarizes results from some existing literature. It is seen that the waveguide crossings we designed have a small footprint. In contrast with other waveguide crossings, the measured insertion loss and crosstalk are relatively low.

**Table 2:** Comparison of the waveguide crossings.

References	Footprint (μm^2^)	IL (dB)	CT (dB)	Method	Note
This work	2.4 × 2.4	0.5	− 19	DBS	Auto-actuated
	2.4 × 3.6	0.56	− 21	DBS	Auto-actuated
^9^	175.2 × 175.2	0.009	< − 37	Self-imaging	Large footprint
^10^	13 × 13	0.4	− 30	Manual adjustment	Multiple experiments
^11^	6 × 6	1.7	− 40	Parabolic taper	Hand-actuated
^12^	6 × 6	0.2	− 40	GA	20 nm bandwidth
^13^	6 × 6	0.18	− 41	PSO	50 generations
^14^	6 × 6	0.028, 0.017	− 37	Step by step	Only operating at 1550 nm and 1310 nm
^15^	21 × 21	1.82	− 18	Y-junction	Large footprint
^16^	2.4 × 2.4	1.5	− 18	Self-imaging	Requiring two crossings
^17^	1 × 1	0.175	− 30	PSO	500 generations
^18^	4.8 × 4.8	0.6	− 24	Symmetric	Still large

### Optical interconnection integrated structures

In principle, the optical signal can be delivered to any chip locations via any routing path provided the low loss bending and crossing are available. For a proof-of-concept demonstration, we arbitrarily design four optical interconnection integrated structures with the proposed waveguide bend and crossings.

**Figure 4:**
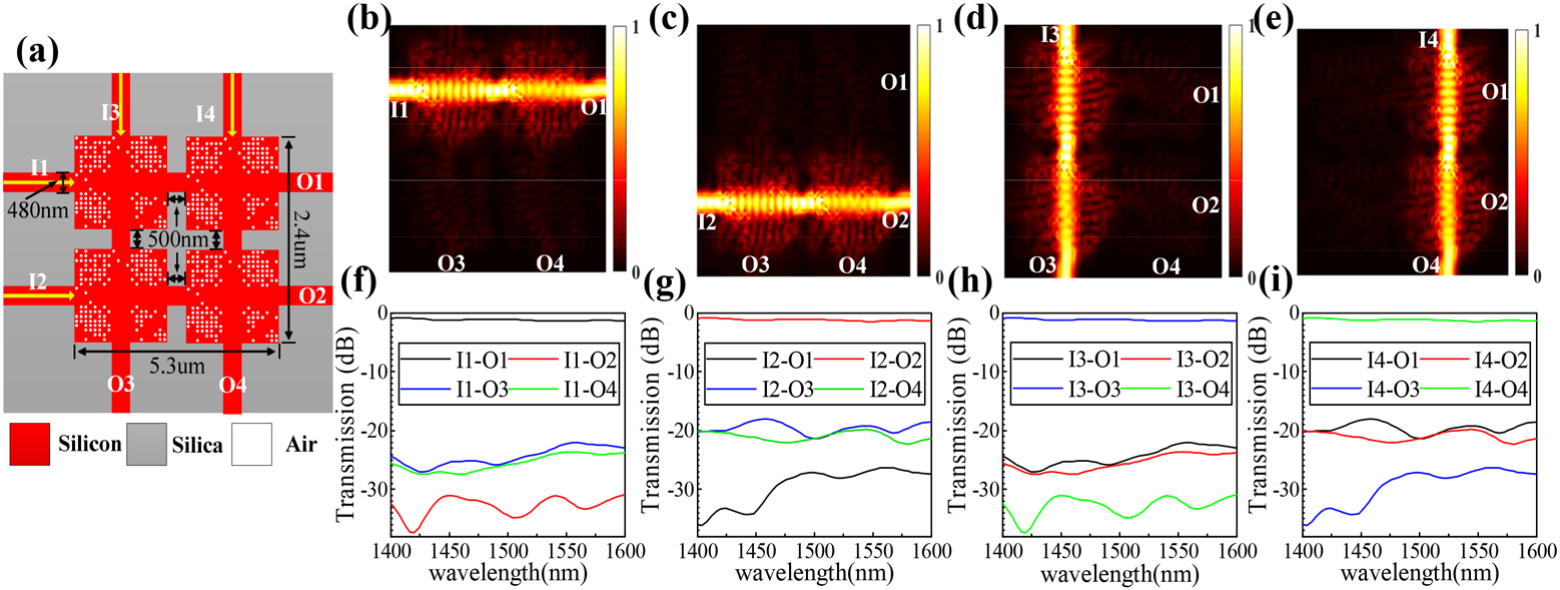
Design and simulation results of the “2_2_2_2” structure. (**a**) Schematic diagram of the structure. (**b**)–(**e**) Simulated optical field distributions. (**f**)–(**i**) Simulated normalized transmission spectra.

The first structure consists of four waveguide crossings, which is named “2_2_2_2” structure. The schematic diagram of this structure is shown in Fig. 4a, its footprint is 2.4 × 5.3 μm^2^, the span between the four crossings is 500 nm, and the input waveguides and the output waveguide are 480 nm. We define input ports I1–I4 and output ports O1‒O4, respectively. Figure 4b–e shows the simulated optical field when the continuous wave is launched from I1-I4 ports, respectively. The match IL and CT spectra are plotted in Fig. 4f–i. The result curves in Fig. 4f,h, and g,i are consistent due to the symmetric structure. The average IL is ~ 1 dB and the CTs are <  − 18 dB within the wavelength range from 1,400 to 1600 nm.

**Figure 5:**
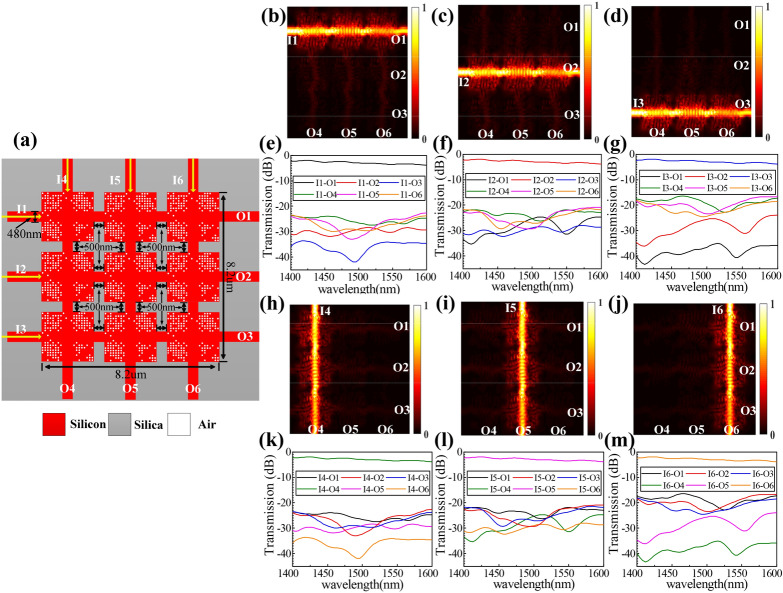
Design and simulation results of the “3_3_3_3” structure. (**a**) Schematic diagram of the structure. (**b**)–(**d**) (**i**)–(**k**) Simulated optical field distributions. (**e**)–(**g**) (**l**)–(**n**) Simulated normalized transmission spectra.

The second structure with a footprint of 8.2 × 8.2 μm^2^ consists of nine waveguide crossings, which is named “3_3_3_3” structure. The relevant specific parameters of this structure are shown in Fig. 5a. We define input ports I1–I6 and output ports O1‒O6, respectively. Figure 5b–d and h–j show the simulated optical field when the continuous wave is launched from I1-I6 ports, respectively. The match IL and CT spectra are plotted in Fig. 5e–g and k–m. The result curves in Fig. 5e and k, f and l, g and m are consistent due to the symmetric structure. The average IL for all output ports is 2.5 dB from 1,400 to 1,600 nm. The CTs are measured to be <  − 16 dB within the same wavelength range.

**Figure 6:**
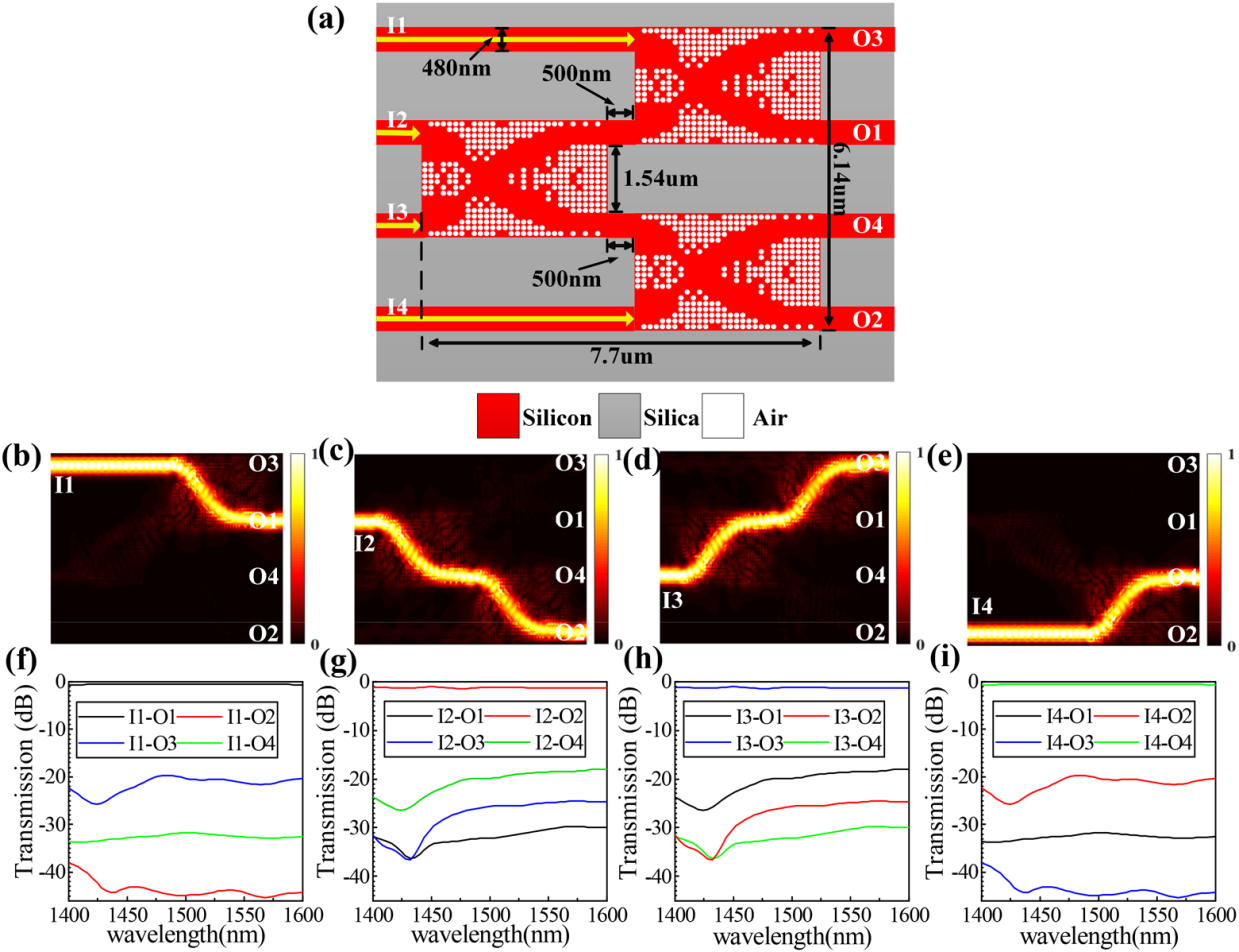
Design and simulation results of the “4_4” structure. (**a**) Schematic diagram of the structure. (**b**)–(**e**) Simulated optical field distributions. (**f**)–(**i**) Simulated normalized transmission spectra.

The third structure with a footprint of 7.7 × 6.14 μm^2^ consists of three same direction waveguide crossings, which is named “4_4” structure. The labels of input ports and output ports and other relevant specific parameters of this structure are shown in Fig. 6a. Figure 6b–e shows the simulated optical field when the continuous wave is launched from I1-I4 ports, respectively. The match IL and CT spectra are plotted in Fig. 6f–i. The result curves in Fig. 6f and i, g and h are consistent due to the symmetric structure. When the input waves from I1 or I4 input ports, the losses come from the one times cross connection, and the average IL is ~ 0.6 dB and the CTs are <  − 20 dB within the wavelength range from 1,400 to 1600 nm. When the input waves from I2 or I3 input ports, the losses come from the two times cross connection, and the average IL is ~ 1.2 dB from 1,400 to 1,600 nm, the CTs are measured to be <  − 16 dB within the same wavelength range.

**Figure 7:**
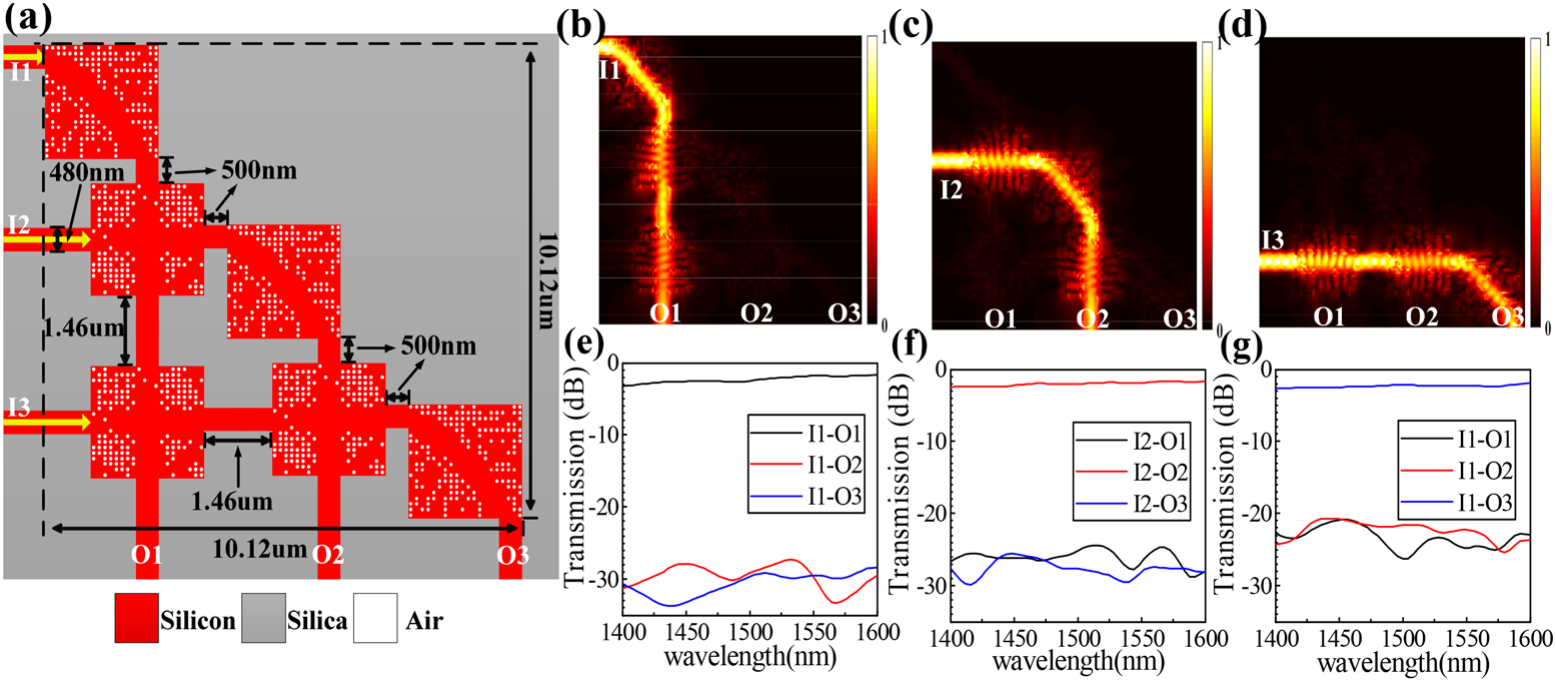
Design and simulation results of the “3_3” structure. (**a**) Schematic diagram of the structure. (**b**)–(**d**) Simulated optical field distributions. (**e**)–(**g**) Simulated normalized transmission spectra.

The fourth structure with a footprint of 10.12 × 10.12 μm^2^ consists of three waveguide bends and three waveguide crossing, which is named “3_3” structure. The relevant specific parameters of this structure are shown in Fig. 7a. We define input ports I1–I3 and output ports O1‒O3, respectively. Figure 7b–d shows the simulated optical field when the continuous wave is launched from I1-I3 ports, respectively. The match IL and CT spectra are plotted in Fig. 7e–g. The average ILs for O1, O2 and O3 output ports are ~ 2.5 dB, ~ 2 dB and ~ 3 dB from 1,500 to 1,580 nm, respectively. The CTs are for O1, O2 and O3 output ports measured to be <  − 27 dB, <  − 25 dB and <  − 21 dB within the same wavelength range, respectively.

The above is a few examples of optical interconnect structures using the waveguide bend and crossings. It is obviously that the number of devices contributes to the losses of the structures. The more devices we use, the greater the losses. The minimum intervals between all devices is 500 nm making sure there is not any out-of-plane light coupling.

## Discussion

In this paper, we use inverse design concept and DBS algorithm to design ultra-compact high efficiency and low crosstalk waveguide bend and crossings for optical interconnection based on SOI platform. The footprints of them are only 2.4 × 2.4 μm^2^, 2.4 × 2.4 μm^2^ and 2.4 × 3.6 μm^2^ respectively. The waveguide bend realizes the 90 degree bending of light, up to 0.18 dB transmission efficiency. The ILs and CTs of the waveguide crossings are less than 0.5 dB, 0.56 dB, − 19 dB and − 21 dB, respectively. Furthermore, we arbitrarily design four optical interconnection integrated structures with the proposed waveguide bend and crossings. All of them exhibit high performances. Actually, more optical interconnect structures can be achieved to deliver optical signal using them. The ultra-compact structures will significantly improve the integration density and benefit various on-chip optical systems. Meantime, our design thought and algorithm flow can also be applied to the research and design of other on-chip photonic devices, such as the optical power beam splitter, the mode division multiplexer, the wavelength division multiplexer, etc.

### Design method

The overall design idea of inverse design: Firstly, setting the target performance of the devices. Then, according to the set performance requirements, the devices are designed and optimized by using various optimization algorithms. Here, we use DBS algorithm to design the devices. The general process is as follows:

1. Dividing pixels: The optimized region is divided into a certain number of pixels. The geometry of each pixel can be circle, square, or other complex shapes. The minimum feature size can be determined according to the fabrication capability. In this paper, each pixel is a square of 120 nm × 120 nm with a central circular hole. The hole has a diameter of 90 nm.
2. Establishing the initial pixel states: Each pixel can occupy two states: silicon or air. For the initial pattern of the devices, all the pixel states are chosen to be silicon in this paper.
3. Defining figure-of-merit (FOM): the FOM is used to see if the pixel state is witched. Different FOMs are defined according to different devices. In this paper, the FOM is defined as Eqs. (1) or (2).
4. Witching the pixel state: the state of each pixel is altered one by one, and the FOM is inspected. The pixel state is retained if the FOM is improved. If not, the pixel state is reversed and the algorithm proceeds to the next pixel. One iteration ends after all the pixel states are inspected.
5. Iterative refinement: The iterations continue until the FOM does not improve further.

## Supplementary information

Supplementary Information.

Supplementary Video S1.

Supplementary Video S2.

Supplementary Video S3.

## Data Availability

All data generated or analyzed during this study are included in this article.
