# Supplementary material for: Ultra-compact high efficiency and low crosstalk optical interconnection structures based on inverse designed nanophotonic elements
Source: Sci Rep. 2020 Jul 20;10:11993. doi: 10.1038/s41598-020-68936-w (PMC7371873; doi:10.1038/s41598-020-68936-w)
Supplement: Supplementary file 1 — Supplementary Information. [file 41598_2020_68936_MOESM1_ESM.docx]

Ultra-compact high efficiency and low crosstalk optical interconnection structures based on inverse designed nanophotonic elements

**Zikang Li, Guofeng Li, Jie Huang, Zhenrong Zhang, Junbo Yang, Changming Yang, Yang Qian, Wenjie Xu and Huimin Huang**

**Supplementary Video**

The supplementary videos visualize the electromagnetic energy density,

*U* = ϵ|**E**|^2^ + μ|**H**|^2^ (1)

in a horizontal slice through the middle of the device as a function of wavelength. Here, ϵ is the permittivity, µ is the magnetic permeability, and **E** and **H** are the **E**-and **H**-fields respectively. The wavelength is displayed in the bottom left corner of the videos.
